# A reverse transcription-quantitative polymerase chain reaction system for evaluating intestinal butyrate production by fecal bacteria

**DOI:** 10.1128/aem.01836-25

**Published:** 2025-12-12

**Authors:** Mai Hane, Kazuhito Shimamoto, Ryo Matsui, Kensuke Shimizu, Miyuki Katto, Takashi Asahara, Yoshiyuki Shishido, Takashi Kurakawa

**Affiliations:** 1 Yakult Central Institute, Yakult Honsha Co., Ltd. 74051 , Kunitachi, Tokyo, Japan; Universita degli Studi di Napoli Federico II, Portici, Italy

**Keywords:** butyryl-CoA:acetate CoA-transferase, butyrate, qPCR, RT-qPCR, feces, synbiotics, microbiota

## Abstract

Intestinal butyrate is key in maintaining host health. As an indicator of butyrate production, the fecal butyrate concentration is used for assessing health status; however, it may not be a suitable marker as it only accounts for residual fecal butyrate following intestinal absorption. Therefore, we aimed to develop a novel quantitative system for evaluating intestinal butyrate production. We designed a primer set targeting the gene encoding butyryl-coenzyme A (CoA):acetate CoA-transferase (*but*), a key enzyme involved in butyrate synthesis. The new primer set specifically reacted with *but*-harboring bacteria, including *Faecalibacterium*, suggesting its suitability for the comprehensive and accurate detection of intestinal butyrate-producing bacteria. We established a system for measuring *but* mRNA levels using the novel primer set and found that the *but* expression levels were more closely correlated with butyrate production than *but* DNA copy number in an *in vitro* culture model. Finally, we verified the effects of synbiotics by fecal analysis based on this primer set and found that synbiotics significantly increased *but* expression levels, but not DNA copy number. In conclusion, our *but* expression quantification system based on a novel primer set is a promising tool for evaluating butyrate production and verifying the efficacy of probiotics and prebiotics.

**IMPORTANCE:**

Intestinal butyrate has diverse functions, including intestinal barrier function enhancement and anti-inflammatory effects, and has therapeutic potential for metabolic and neurological health. Butyrate affects human health, and intestinal butyrate production must be accurately measured for understanding health status. However, as over 95% of the butyrate produced by intestinal bacteria is absorbed in the intestinal tract, direct fecal butyrate concentration measurement may not accurately reflect the amount produced by intestinal bacteria. Herein, we developed a system for measuring the expression levels of butyrate-producing genes in intestinal bacteria and demonstrated its suitability for estimating butyrate production capacity. This system can be used in the development of probiotics, prebiotics, and synbiotics. As butyrate-based preventive and therapeutic methods for various diseases are developed in the future, this system will aid in verifying their effectiveness and elucidating the mechanisms of action.

## INTRODUCTION

The large intestines in humans harbor over 1,000 different types of bacteria, amounting to ~100 trillion bacterial cells (1). Metabolites produced by these intestinal bacteria, such as short-chain fatty acids (SCFAs), putrefactive products, amines, secondary bile acids, and vitamins, significantly affect host health (2, 3). SCFAs, including acetate, propionate, and butyrate, are major beneficial metabolites produced by gut bacteria via the degradation of indigestible dietary fibers (4). Butyrate is a major energy source utilized predominantly by colonocytes (5, 6). The rapid absorption and utilization of butyrate by epithelial cells increases oxygen consumption, thereby promoting the maintenance of an anaerobic environment in the gut and preventing colonization by opportunistic aerobic pathogens, such as *Salmonella* and *Escherichia coli* (7). Furthermore, butyrate exerts diverse physiological effects, including anti-inflammatory effects, homeostasis of the intestinal immune system, enhancement of intestinal barrier function, and protection against colorectal cancer (7–10), and is expected to have therapeutic effects on metabolic and immunological health (9). Thus, as intestinal butyrate profoundly impacts human health, accurately measuring intestinal butyrate production is essential for understanding human health status and developing therapeutic approaches (9).

Intestinal butyrate production has traditionally been assessed by quantifying the fecal butyrate content using techniques such as high-performance liquid chromatography (HPLC). However, because 95% of intestinal SCFAs are absorbed in the colon (11–14) and only the remaining 5% is detected in excreted feces, this method may not accurately capture the total amount produced by gut bacteria. Patients with hypertension have considerably lower levels of butyrate-producing bacteria compared with healthy individuals, whereas no such trend has been observed in fecal butyrate concentrations (15). This suggests a discrepancy between butyrate production in the gut and its concentration in feces. We considered that focusing on gut butyrate-producing bacteria is a practical approach for evaluating intestinal butyrate production.

In the phylogenetic classification based on 16S rDNA sequences, human intestinal butyrate-producing bacteria are distributed over a very wide range, including *Clostridium* clusters I, IV, IX, X, XI, XIVa, and XVI, belonging to the Bacillota phylum (formerly known as Firmicutes) and the *Fusobacteriaceae* family (16). Among these, *Clostridium* clusters IV and XIVa are predominant in the human intestines (17). In particular, *Faecalibacterium prausnitzii* in cluster IV and *Agathobacter rectalis* (formerly *Eubacterium rectale*), *Anaerostipes*, and *Roseburia* in cluster XIVa are representative dominant groups and species (18). The most common pathway for butyrate production in these bacteria is the acetyl-coenzyme A (CoA) pathway (19). Either butyryl-CoA:acetate CoA-transferase (But) or butyrate kinase (Buk) is involved in the final step of this metabolic pathway from butyryl-CoA to butyrate (19, 20). In contrast to the Buk-mediated pathway, which is present in only a few gut bacteria, the But-mediated pathway is predominant in human colonic ecosystems (21, 22). Furthermore, acetate is required as a substrate in addition to butyryl-CoA, and regenerated acetyl-CoA acts as the substrate for the upstream reaction in the acetyl-CoA pathway; this may be particularly advantageous in colonic ecosystems with high acetate levels (23). These findings indicate that the butyryl-CoA:acetate CoA-transferase-encoding gene (*but*) is a promising biomarker for detecting the main butyrate-producing bacteria in the human colon. Several *but* primers have been reported in previous studies (24–26), with all of them used only to measure *but* copy abundance through the quantitative polymerase chain reaction (qPCR). However, we hypothesized that *but* expression levels would be a valuable parameter for evaluating butyrate production status in the intestines.

It has been demonstrated through *in vitro* gut model that probiotics, prebiotics, and synbiotics (a combination of probiotics and prebiotics) are effective in increasing butyrate production (27–29). In particular, synbiotics are expected to have a synergistic effect on adjusting the gut microbiota and increasing SCFA levels (30). Specific synbiotics, consisting of *Lacticaseibacillus paracasei* strain Shirota (formerly *Lactobacillus casei* strain Shirota, LcS), *Bifidobacterium breve* strain Yakult (BbrY), and galacto-oligosaccharides, have been shown in multiple randomized controlled trials (RCTs) to improve intestinal SCFA levels, including butyrate, in disease patients (31–35). However, the effects of this synbiotics on healthy adults have not yet been investigated.

In this study, we aimed to enable rapid and simple evaluation of intestinal butyrate production capacity. We constructed a single primer pair capable of amplifying a wide range of *but* and developed a novel quantitative system for determining *but* expression by reverse transcription (RT)-qPCR. We also applied this system to fecal samples of healthy adults to assess its usefulness as a tool for estimating butyrate production capacity in the gut and verifying the efficacy of synbiotics.

## RESULTS

### Newly designed primer set exhibited excellent specificity

**Fig 1:**
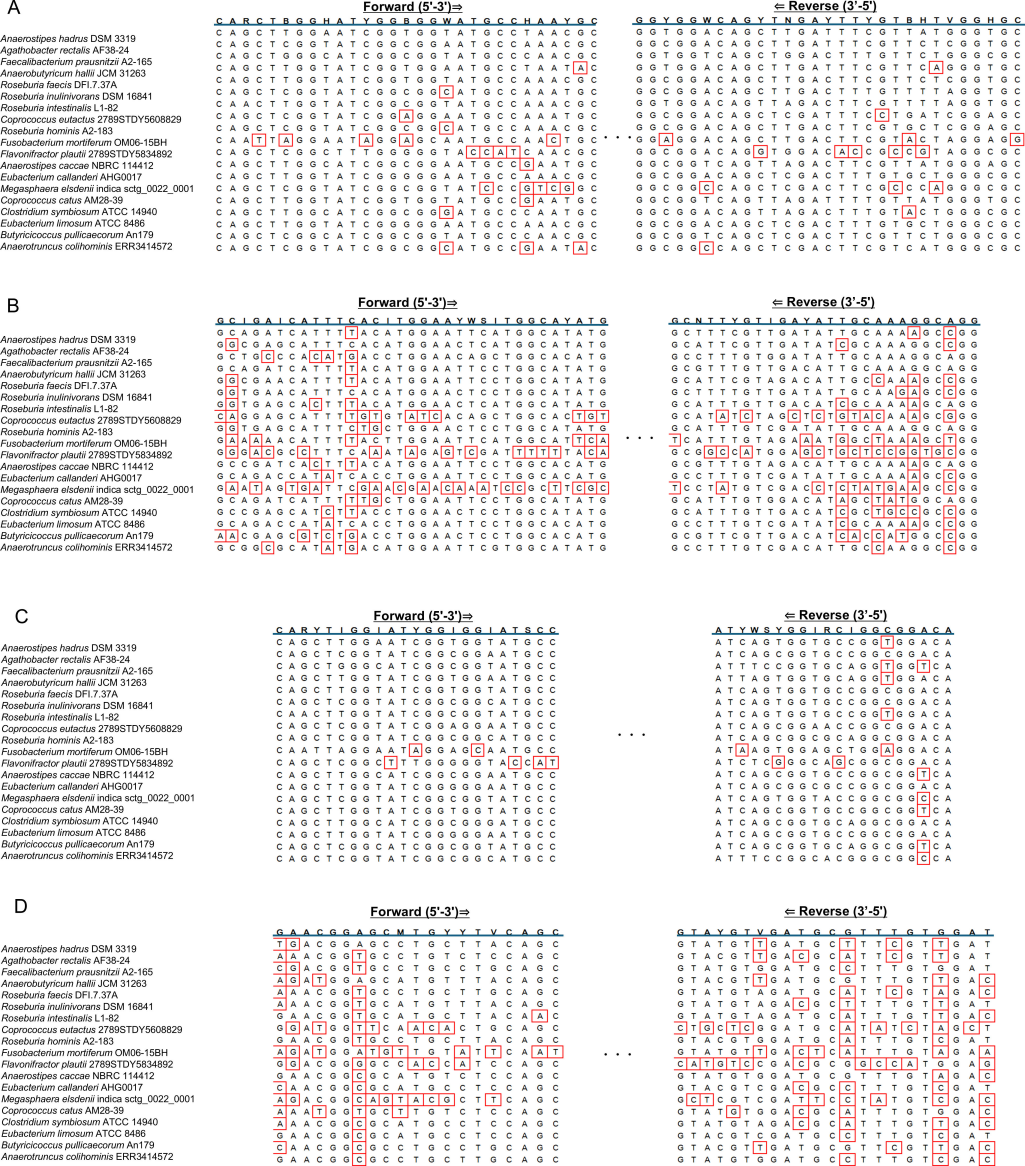
Alignment results for primer sequences and target regions of *but*-harboring strains. (**A**) but_652F3/but_1025R3, (**B**) Flint et al. (24), (**C**) Trachsel et al. (25), and (**D**) Wang et al. (26). Mismatched regions within the primer sequence are enclosed in red boxes. R: A/G, M: A/C, W: A/T, S: C/G, Y: C/T, K: G/T, H: A/T/C, B: G/T/C, D: G/A/T, V: A/C/G, N: A/C/G/T, I: deoxyinosine.

We evaluated the specificity of the newly designed primer set, as well as that of previously published primer sets. Assessment of the alignment of each primer sequence, as well as that of the primer target region of the representative *but*-harboring strains, showed that the primer sets published by Louis and Flint (24) and Wang et al. (26) exhibited higher numbers of mismatches than the newly designed primer set (Fig. 1). In contrast, we observed only a few mismatches in the primers published by Trachsel et al. (25). This could be attributed to the frequent use of deoxyinosine (I), which binds to any base (Fig. 1).

Of the 20 *but*-harboring strains confirmed to produce butyrate, the newly designed primer set reacted with DNA from 16 strains, except for those from *Coprococcus catus* YIT 11484^T^, *Fusobacterium mortiferum* YIT 10361^T^, *Flavonifractor plautii* YIT 12796^T^, and *Anaerotruncus colihominis* JCM 15631 (Table 1). Reactivity to *Coprococcus catus* YIT 11484^T^ was relatively weak. This primer set did not amplify the 64 non-*but*-harboring strains (Table 1). There was a 1,000-fold or greater difference in reactivity between the target and non-target species. In contrast, previously published primers showed low coverage for *but*-harboring bacteria (Table 1). For example, none of the primer sets reacted with *Faecalibacterium prausnitzii* YIT 10067^T^, a representative butyrate-producing human gut bacterium. The primers published by Trachsel et al. (25) and Wang et al. (26) were confirmed to react non-specifically with non-*but*-harboring bacteria. These results showed that the newly designed primer set was more specific to *but* than previously published primers.

**TABLE 1:** Primer set specificity tests for *but* detection

Taxon	Strain	Butyrate production^*a*^	Butyrate synthesis gene		Reaction^*c*^			
			*but*	Other^*b*^	This study	Flint et al.	Trachsel et al.	Wang et al.
*Roseburia faecis*	YIT 11921^T^	+	+	−	+	+	+	+
*Roseburia intestinalis*	YIT 10172^T^	+	+	−	+	+	+	+
*Roseburia hominis*	JCM 17582	+	+	−	+	+	+	+
*Butyricicoccus pullicaecorum*	YIT 12785^T^	+	+	−	+	+	+	+
*Anaerostipes hadrus*	YIT 13225	+	+	+	+	+	+	+
*Agathobacter rectalis*	YIT 6082^T^	+	+	+	+	+	+	−
*Faecalibacterium prausnitzii*	YIT 10067^T^	+	+	+	+	−	−	−
*Anaerobutyricum hallii*	YIT 10064^T^	+	+	+	+	+	+	+
*Roseburia inulinivorans*	YIT 11922^T^	+	+	+	+	+	+	+
*Coprococcus eutactus*	YIT 10160^T^	+	+	+	+	−	+	−
*Anaerostipes caccae*	YIT 10168^T^	+	+	+	+	+	−	+
*Eubacterium callanderi*	YIT 10175^T^	+	+	+	+	+	+	−
*Megasphaera elsdenii*	YIT 6063^T^	+	+	+	+	−	+	−
*Clostridium symbiosum*	YIT 11480^T^	+	+	+	+	−	+	+
*Eubacterium limosum*	YIT 6067^T^	+	+	+	+	−	+	+
*Anaerostipes butyraticus*	YIT 12362^T^	+	+	+	+	+	+	+
*Coprococcus catus*	YIT 11484^T^	+	+	+	−	−	+	−
*Fusobacterium mortiferum*	YIT 10361^T^	+	+	+	−	−	−	−
*Flavonifractor plautii*	YIT 12796^T^	+	+	+	−	−	−	−
*Anaerotruncus colihominis*	JCM 15631	+	+	+	−	−	−	−
*Blautia luti*	YIT 12257^T^	−	−	−	−	−	+	+
*Bifidobacterium pseudocatenulatum*	YIT 4072^T^	−	−	−	−	−	−	−
*Bifidobacterium adolescentis*	YIT 4011^T^	−	−	−	−	−	−	−
*Bacteroides vulgatus*	YIT 6159^T^	−	−	−	−	−	−	+
*Prevotella copri*	YIT 12933^T^	−	−	−	−	−	−	+
*Ruminococcus bromii*	YIT 6078	−	−	−	−	−	−	−
*Akkermansia muciniphila*	YIT 11774^T^	−	−	−	−	−	−	−
*Parabacteroides distasonis*	YIT 12678	−	−	−	−	−	−	+
*Bifidobacterium breve*	YIT 4014^T^	−	−	−	−	−	−	−
*Bifidobacterium bifidum*	YIT 4039^T^	−	−	−	−	−	−	−
*Eggerthella lenta*	YIT 6077^T^	−	−	−	−	−	−	−
*Eubacterium siraeum*	YIT 10049^T^	−	−	−	−	−	−	−
*Bifidobacterium dentium*	YIT 4017^T^	−	−	−	−	−	−	−
*Bifidobacterium animalis*	YIT 4044^T^	−	−	−	−	−	−	+
*Veillonella atypica*	YIT 6081^T^	−	−	−	−	−	−	+
*Streptococcus mutans*	YIT 2026^T^	−	−	−	−	−	−	+
*Bifidobacterium angulatum*	YIT 4012^T^	−	−	−	−	−	−	−
*Lactobacillus gasseri*	YIT 0192^T^	−	−	−	−	−	−	−
*Streptococcus anginosus*	YIT 11237^T^	−	−	−	−	−	−	+
*Raoultella planticola*	YIT 10131^T^	−	−	−	−	−	−	−
*Veillonella parvula*	YIT 6072^T^	−	−	−	−	−	−	−
*Clostridium asparagiforme*	YIT 12840^T^	−	−	−	−	−	−	−
*Clostridium hylemonae*	YIT 12258^T^	−	−	−	−	−	−	+
*Bifidobacterium pseudolongum*	YIT 4102^T^	−	−	−	−	−	−	−
*Bifidobacterium gallinarum*	YIT 4094^T^	−	−	−	−	−	−	−
*Citrobacter amalonaticus*	YIT 10116^T^	−	−	−	−	−	−	+
*Atopobium minutum*	YIT 0194	−	−	−	−	−	−	−
*Enterobacter cloacae*	YIT 6041^T^	−	−	−	−	−	−	−
*Ruminococcus torques*	YIT 10159^T^	−	−	−	−	−	−	−
*Bifidobacterium catenulatum*	YIT 4072^T^	−	−	−	−	−	−	−
*Dialister succinatiphilus*	YIT 11850^T^	−	−	−	−	−	−	+
*Bifidobacterium longum*	YIT 4021^T^	−	−	+	−	−	−	−
*Collinsella aerofaciens*	YIT 10235^T^	−	−	+	−	−	−	−
*Bacteroides uniformis*	YIT 6164^T^	−	−	+	−	−	−	+
*Ruminococcus gnavus*	YIT 6176^T^	−	−	+	−	−	−	+
*Bacteroides stercoris*	YIT 12663	−	−	+	−	−	−	+
*Bacteroides fragilis*	YIT 6158^T^	−	−	+	−	−	−	+
*Bacteroides ovatus*	YIT 6161^T^	−	−	+	−	−	−	+
*Phocaeicola plebeius*	YIT 12661	−	−	+	−	−	−	−
*Bacteroides thetaiotaomicron*	YIT 6163^T^	−	−	+	−	−	−	+
*Erysipelatoclostridium ramosum*	YIT 10062^T^	−	−	+	−	−	−	−
*Alistipes onderdonkii*	YIT 12691	−	−	+	−	−	−	−
*Enterococcus avium*	YIT 10255^T^	−	−	+	−	−	−	+
*Enterocloster clostridioformis*	YIT 6051^T^	−	−	+	−	−	+	+
*Alistipes finegoldii*	YIT 12685	−	−	+	−	−	−	−
*Alistipes indistinctus*	YIT 12060^T^	−	−	+	−	−	−	−
*Clostridium scindens*	YIT 6171^T^	−	−	+	−	−	−	−
*Blautia producta*	YIT 6141^T^	−	−	+	−	−	−	+
*Escherichia coli*	YIT 6044^T^	−	−	+	−	−	−	+
*Clostridium citroniae*	YIT 12646^T^	−	−	+	−	−	+	+
*Enterococcus faecalis*	YIT 2031^T^	−	−	+	−	−	−	−
*Citrobacter freundii*	YIT 6045^T^	−	−	+	−	−	−	+
*Olsenella uli*	YIT 12014^T^	−	−	+	−	−	−	−
*Eubacterium ventriosum*	YIT 10066^T^	+	−	+	−	−	−	−
*Coprococcus comes*	YIT 12793^T^	+	−	+	−	−	−	+
*Eubacterium ramulus*	YIT 12128^T^	+	−	+	−	−	−	+
*Odoribacter splanchnicus*	YIT 12675^T^	+	−	+	−	−	+	+
*Fusobacterium varium*	YIT 12723	+	−	+	−	−	−	−
*Citrobacter koseri*	YIT 10117^T^	−	−	+	−	−	+	+
*Acidaminococcus fermentans*	YIT 6071^T^	+	−	+	−	−	−	−
*Subdoligranulum variabile*	YIT 12797^T^	+	−	+	−	−	−	−
*Fusobacterium necrophorum subsp. necrophorum*	YIT 10343^T^	+	−	+	−	−	−	−
*Fusobacterium nucleatum subsp. nucleatum*	YIT 6069^T^	+	−	+	−	−	−	−
*Fusobacterium periodonticum*	YIT 12430^T^	+	−	+	−	−	−	−

^*a*^ Presence or absence of butyrate production in culture. +: above the lower limit of detection (1.7 μmol/mL). −: below the lower limit of detection.

^*b*^ Butyrate-encoding genes: *buk* (butyrate kinase), *AtoA*/*D* (acetate CoA/acetoacetate CoA-transferase A/D), *4Hbt* (4-hydroxybutyrate CoA-transferase).

^*c*^ Specificity assays were performed using the newly designed primer set, previously published primer sets, and DNA extracts corresponding to 10^5^ copies from each described strain. +: >10^4^ standard copies per reaction. −: <10^4^ standard copies per reaction.

### *but* expression levels reflected the amount of butyrate produced by butyrate-producing bacteria

We established a system for measuring *but* mRNA levels using the novel primer set. *but* RNA counts were well correlated with the threshold cycle (*C_q_*) values obtained through RT-qPCR (Fig. S1, *R*^2^ > 0.99). The lower detection limit for *but* RNA was 10^2^ copies/reaction (Fig. S1).

**Fig 2:**
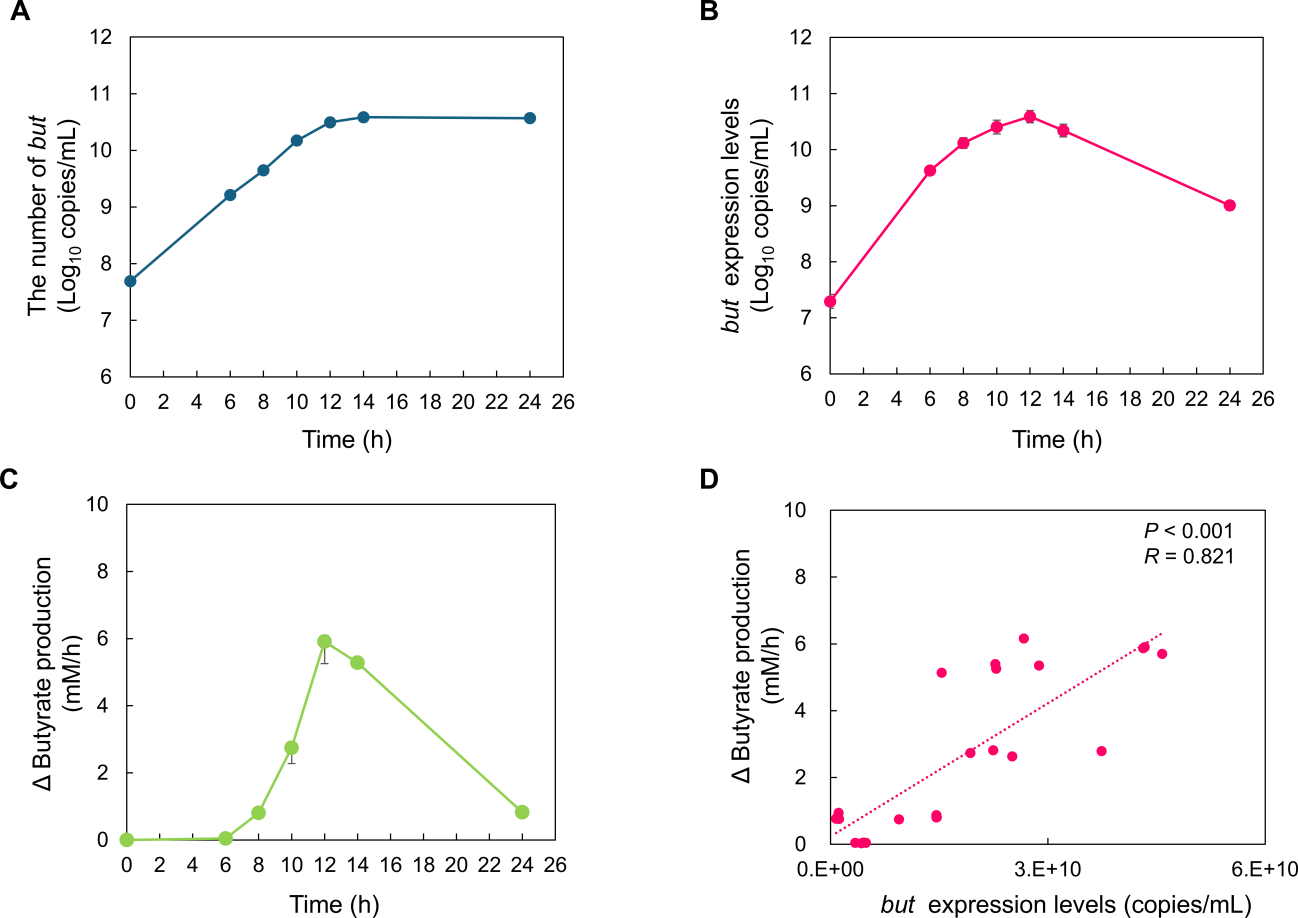
Correlation between *but* copy number and expression levels, and butyrate production per hour in the *in vitro* culture system. *but* copy numbers (**A**), *but* expression levels (**B**), and butyrate production per hour (Δbutyrate production) (**C**) following mixed culture. (**D**) Correlation between *but* expression levels and butyrate production per hour. Values are presented as mean ± SD (*n* = 4).

*but* copy numbers and expression levels, and butyrate concentrations were determined and compared over time following a mixed culture of three *but-*harboring strains (*Anaerostipes hadrus* YIT 13225, *Anaerobutyricum hallii* YIT 10064^T^, and *Roseburia intestinalis* YIT 10172^T^) and one non-*but*-harboring strain (*Prevotella copri* YIT 12933^T^). The *but* copy number determined through qPCR increased until 12 h of culture, following which it plateaued (Fig. 2A). In contrast, the *but* expression levels determined through RT-qPCR increased until 12 h of culture and decreased until 24 h of culture (Fig. 2B).

The amount of butyrate produced *in vitro* per hour (Δbutyrate production) by *but*-harboring bacteria increased over time up to 12 h of culture and then decreased until 24 h of culture (Fig. 2C).

The trend in Δbutyrate production resembled that in *but* expression and differed from that in *but* copy number. A significant positive correlation was observed between Δbutyrate production and *but* expression (Fig. 2D, *P* < 0.001, *R* = 0.821).

The viable cell count measured using RT-qPCR targeting 16S rRNA increased over time up to 12 h of culture and then decreased until 24 h of culture (Fig. S2).

### Application of *but* quantification system for evaluating the effects of synbiotics

The *Bifidobacterium* and total lactobacilli fecal levels in healthy adults increased during synbiotic intake compared with those at 0 weeks (*P* < 0.05 and *P* < 0.01, respectively; Table S1). In contrast, the *Clostridium perfringens* levels were significantly lower at 2 weeks than at 0 weeks (*P* < 0.05; Table S1). After synbiotic intake discontinuation, *Enterococcus* levels significantly decreased compared with those observed at 2 weeks (*P* < 0.05; Table S1). In all participants, LcS and BbrY were detected at levels of 10^7^ and 10^6^ cells/g of feces at 1 and 2 weeks, respectively (Table S1).

**Fig 3:**
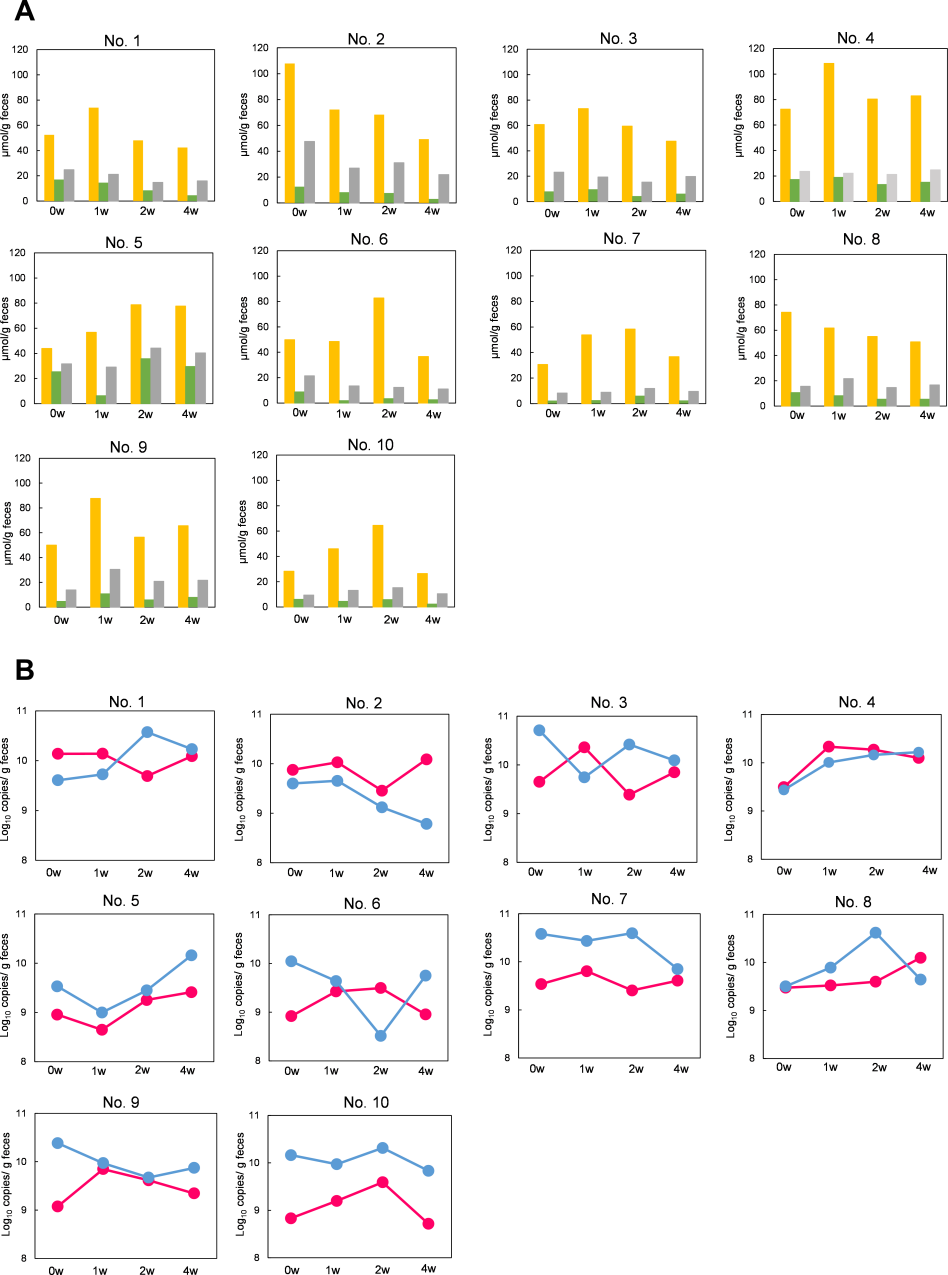
Monitoring of changes in individual fecal SCFA concentrations and *but* copy number and expression levels during the synbiotic intake period. (**A**) Acetate (yellow), butyrate (green), and propionate (gray) concentrations in feces. (**B**) Changes in fecal *but* copy number (blue) and *but* expression levels (pink).

Monitoring individual changes revealed an increase in fecal acetate concentrations in 8 of the 10 participants (1, 3, 4, 5, 6, 7, 9, and 10) during synbiotic intake (Fig. 3). Slight changes in the butyrate and propionate concentrations were observed during the intake period for participants 2 and 5; however, the majority of participants did not exhibit any fluctuations (Fig. 3). In contrast to fecal butyrate concentrations, *but* copy numbers and expression levels were variable in response to stimuli. Except for participants 4, 5, and 10, *but* copy number and expression levels showed different trends for all participants; in addition, *but* expression levels increased in many individuals during the synbiotic intake period (Fig. 3). Eight (2, 3, 4, 6, 7, 8, 9, and 10) and six (4, 5, 6, 8, 9, and 10) participants exhibited higher *but* expression levels at 1 and 2 weeks, respectively (Fig. 3), than at 0 weeks.

Regarding overall changes in the 10 healthy adults, fecal acetate concentration tended to increase at 1 week compared with that at 0 weeks (*P* = 0.106) and significantly decreased at 4 weeks compared with that at 2 weeks (*P* < 0.05). Fecal butyrate and propionate concentrations did not change significantly during or after synbiotic intake (Table 2). Furthermore, there was no significant change in *but* copy number in feces before and after synbiotic intake; however, there was a significant increase in *but* expression levels at 1 and 4 weeks compared with those at 0 weeks (Table 3, *P* < 0.05).

**TABLE 2:** Effect of synbiotic intake on fecal SCFAs^*a*^ and pH

	0 weeks		1 week		2 weeks		4 weeks	
Acetate	57.1 ± 23.5	100	68.3 ± 19.2	100	65.2 ± 12.0	100	51.6 ± 18.4^*c*^	100
Butyrate	11.3 ± 6.9	100	8.6 ± 5.3	100	9.7 ± 9.6	100	8.0 ± 8.5	100
Propionate	22.0 ± 11.6	100	20.7 ± 7.1	100	20.2 ± 10.2	100	19.3 ± 9.1	100
pH	7.2 ± 0.6		6.8 ± 0.5		6.7 ± 0.3^*b*^		7.2 ± 0.7	

^*a*^ Results are expressed as µmol/g of feces (mean ± SD) and detection rate (%).

^*b*^ *P* < 0.05: comparison before and after intake (Wilcoxon signed-rank test).

^*c*^ *P *< 0.05: comparison between 2 and 4 weeks (Wilcoxon signed-rank test).

**TABLE 3:** Effect of synbiotic intake on intestinal *but* copy number and expression levels^*a*^

	0 weeks		1 week		2 weeks		4 weeks	
*but* copy numbers	9.96 ± 0.48	100	9.80 ± 0.37	100	9.94 ± 0.73	100	9.84 ± 0.42	100
*but* expression levels	9.40 ± 0.44	100	9.73 ± 0.54^*b*^	100	9.58 ± 0.28	100	9.63 ± 0.51^*b*^	100

^*a*^ Results are expressed as Log_10_ copies/g of feces (mean ± SD) and detection rate (%).

^*b*^ *P* < 0.05: comparison before and after intake (Wilcoxon signed-rank test).

**Fig 4:**
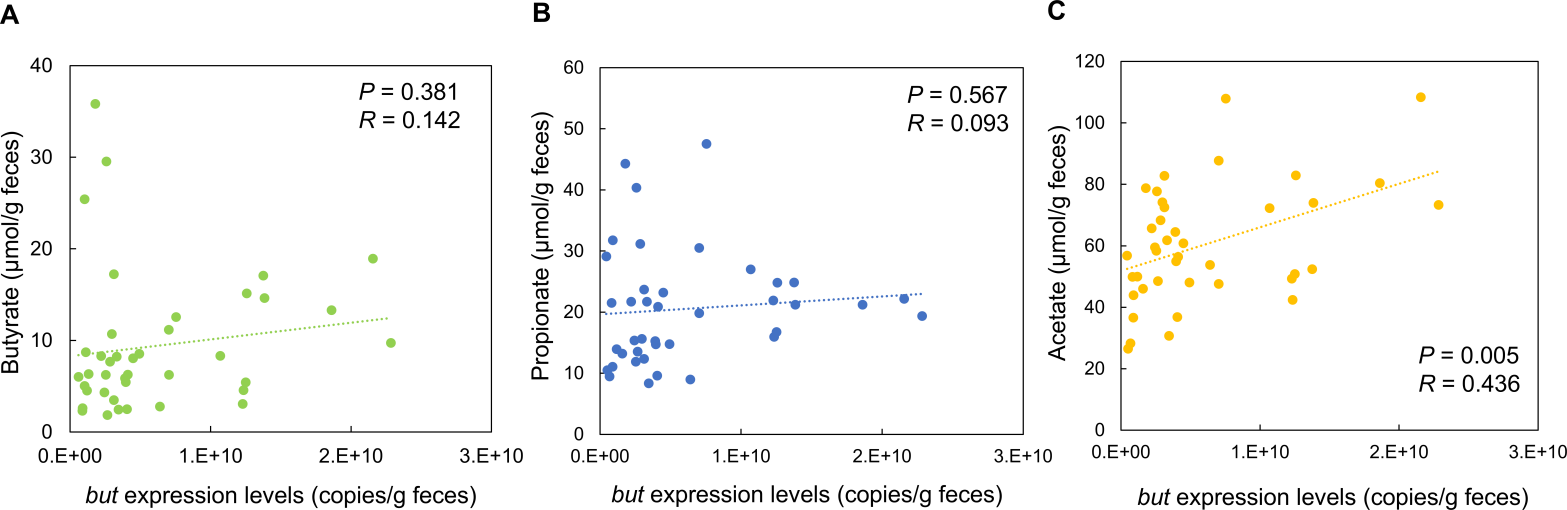
Correlation between fecal *but* expression levels and concentrations of butyrate, propionate, and acetate. Correlation plots between *but* expression levels and fecal butyrate (**A**), propionate (**B**), and acetate (**C**) concentrations for 40 fecal samples from healthy adults.

*but* expression levels were not significantly correlated with butyrate and propionate concentrations (Fig. 4A and B) but positively correlated with fecal acetate concentrations (Fig. 4C, *P* < 0.01, *R* = 0.436). No correlation was observed between *but* copy number and the SCFA concentration (Fig. S3).

## DISCUSSION

Butyrate produced by intestinal bacteria is important for maintaining host health, and the accurate evaluation of its intestinal production status is of great significance (13). In this study, to accurately determine the change in intestinal butyrate content, we focused on *but*, an important functional gene for butyrate production in gut bacteria, and developed a rapid and feasible system for *but* quantification. The quantification of a particular gene of interest (GOI) in a microbial population provides valuable insights into the real functional capacity of a particular sample; however, the variability in GOI sequences among bacteria limits the applicability of this approach (36). Several single primer pairs have been reported for *but* quantification (24–26). However, we found that they are limited by their low coverage for *but*-harboring strains, consistent with expectations based on alignment analysis, or non-specific reactivity with non-*but*-harboring bacteria, potentially owing to the mixed bases used in their sequences. Particularly, none of these primers reacted with *Faecalibacterium prausnitzii*, which is the most abundant butyrate-producing bacterium in fecal samples (approximately 5%–14% relative abundance) (37). Consistent with our findings, previous studies have shown that these primers may result in the underestimation of the abundance of butyrate-producing bacteria in the gut (24, 25). In this study, we aimed to design new primers that could achieve comprehensive coverage of the major intestinal butyrate-producing bacteria and not react with strains without *but*. Of the 20 butyrate-producing and *but-*harboring bacterial species, the newly designed primer set was non-reactive to *Flavonifractor plautii*, *Fusobacterium mortiferum*, and *Anaerotruncus colihominis*. It was relatively weakly reactive to *Coprococcus catus*. However, it could detect other *but*-harboring bacteria, including *Faecalibacterium prausnitzii* and *Agathobacter rectalis* (relative abundance: 3%–15%) (38, 39), *Anaerobutyricum hallii* (relative abundance: approximately 0.6%) (39), and *Roseburia intestinalis* (relative abundance: 0.9%–5.0%) (39, 40). Thus, the new primers were considered suitable for the comprehensive detection of intestinal butyrate-producing bacteria. Furthermore, the primers did not exhibit cross-reactivity with any of the 64 strains without *but*, suggesting their accuracy for quantifying *but*-harboring strains in fecal samples containing a variety of bacteria in comparison with previously reported primers. The newly designed primers are characterized by the absence of deoxyinosine (I), which is present in the previously reported primers, a long primer sequence (29 bases each for forward and reverse primers), and a high annealing temperature. These characteristics are potentially suitable for non-specific amplification suppression.

The previously reported *but* primers were only used for qPCR assays (24–26). As measuring *but* expression levels would provide another representation of butyrate production, RT-qPCR targeting *but* mRNA was established using the new primer set. Previous studies have reported that DNA-targeting qPCR is closely associated with *in vitro* butyrate production (41), consistent with the results from the early stages of culture in our *in vitro* culture system. *In vitro*, measuring *but* copy numbers is crucial for determining the amount of accumulated butyrate. However, during the later stages of culture, corresponding to the stationary and decline phases, the trend in butyrate production per hour was similar to that observed with RT-qPCR targeting *but* mRNA, but not with DNA-targeted qPCR. Butyrate is produced by intestinal bacteria via anaerobic fermentation for energy production (42), and its activity decreases during the stationary phase, when bacterial growth has ceased (23). As DNA-targeting qPCR measures *but* copy numbers derived from both live and dead bacteria, it was considered that the *but* copy number did not fluctuate, even during the stationary phase. The human intestine contains actively growing, dead, and dormant bacteria in coexistence; this is affected by environmental changes, such as dietary intake and antibiotic administration (43, 44). Therefore, the quantification of *but* expression levels is also considered important for estimating intestinal butyrate production capacity. Thus, when analyzing the human intestine, in addition to quantifying *but* copy number, quantifying *but* expression levels aids accurate understanding of butyrate production. This method is expected to serve as a useful tool for clarifying *but* expression behavior, which has not previously been clarified.

RT-qPCR targeting *but* mRNA was applied for the assessment of fecal samples from healthy participants to verify the effects of synbiotic intake. Previous studies have shown that the intake of these synbiotics improves intestinal microbiota and intestinal environment in disease patients, resulting in beneficial effects (31–35). For example, previous RCTs have shown that, during synbiotic intake, fecal SCFA levels, including butyrate, were significantly higher compared with those at the baseline or in the control group (31–35). In our study, however, healthy individuals only showed a slight tendency toward increased fecal acetate concentration. This can be attributed to the effect of intestinal absorption. Most intestinal SCFAs are absorbed in the colon (11–14). In addition, the fecal butyrate concentration has been reported to correlate with the intestinal permeability index (45). Based on this finding, it is expected that patients with a damaged intestinal epithelium would exhibit impaired intestinal absorption and that the fecal concentrations of some SCFAs would strongly reflect their intestinal concentrations, with the opposite expected in healthy subjects. Thus, more specifically, the measurement of residual fecal SCFA concentrations may not accurately capture changes in intestinal SCFA levels in healthy subjects. However, the *but* expression levels fluctuated dynamically and increased significantly during the synbiotic intake period. The *but* expression measurement system accurately captured changes in butyrate production without being affected by intestinal absorption.

In our intervention study, the *but* expression levels were positively correlated with fecal acetate concentrations. Acetate is produced in higher amounts in the intestines than butyrate and propionate; therefore, it is less affected by intestinal absorption. Acetate is also a source of acetyl-CoA, an important precursor of butyrate in the acetyl-CoA pathway (22, 23). Several butyrate-producing bacteria are net acetate consumers, and an increase in acetate concentration promotes butyrate production in *but*-harboring strains (23, 46). Therefore, intestinal acetate potentially induced an increase in *but* expression levels, resulting in a positive correlation between them. During synbiotic intake, acetate is actively produced upon the administration of BbrY and endogenous *Bifidobacterium*, which can utilize galacto-oligosaccharides (47–49). Lactate is also actively produced by LcS (50) and converted to acetate, propionate, and butyrate by obligate anaerobic bacteria (51). We hypothesized that the increase in *but* expression following synbiotic intake can be attributed to an increase in intestinal acetate concentrations occurring through this mechanism. Our quantitative system for measuring *but* expression, which focuses on intestinal butyrate production, is a novel and promising approach for verifying the effects of product intake and screening materials that can induce butyrate production.

A limitation of this study is the small number of fecal samples used to validate the usefulness of the quantitative system and the lack of a control group or long-term synbiotic intervention. In the future, this primer set should be used in various human studies, including those involving larger-scale human studies and clinical patient samples, to validate its usefulness. Another limitation of this study is that we could not demonstrate whether fecal *but* mRNA reflects the RNA levels from the upper colon, where SCFAs are actively produced. Future validation using human intestinal contents is necessary. Comparing existing technologies, such as metagenomics and RNA sequencing, with this quantitative system and enabling their appropriate selection based on specific objectives is also an important challenge for the future.

In conclusion, we constructed a reliable quantification system, used it to accurately assess butyrate production in the intestines, and demonstrated its applicability. Unlike the conventional methods used for fecal butyrate concentration measurement, this quantitative system can be a useful tool for verifying efficacy from a new perspective. This system can be utilized for the development of probiotics, prebiotics, and synbiotics, as well as for revealing the previously unknown importance of butyrate in disease and its recovery process. As therapies and functional foods based on butyrate are developed for numerous diseases in the future, this system is expected to aid in verifying their efficacy and reactivity, as well as elucidating their mechanisms of action.

## MATERIALS AND METHODS

### Bacterial strains and culture conditions

The bacterial strains and culture conditions are listed in Table S2. Frozen stock of each strain was inoculated into each medium, incubated at 37°C for 16–48 h for preculture, and transferred to the same fresh medium and incubated for 16–48 h.

### Development of butyryl-CoA:acetate CoA-transferase gene-specific primers

*but* DNA sequences for *Anaerostipes hadrus* DSM 3319 (accession no. AMEY01000089), *Agathobacter rectalis* AF38-24 (accession no. NZ_QRON01000006), *Faecalibacterium prausnitzii* (currently, *Faecalibacterium duncaniae*) A2-165 (accession no. CP022479), *Anaerobutyricum hallii* JCM 31263 (accession no. NZ_BLYK01000045), *Roseburia faecis* DFI.7.37A (accession no. NZ_JAJCJO010000006), *Roseburia inulinivorans* DSM 16841 (accession no. ACFY01000152), *Roseburia intestinalis* L1-82 (accession no. ABYJ02000099), *Coprococcus eutactus* 2789STDY5608829 (accession no. CYYZ01000002), *Roseburia hominis* A2-183 (accession no. CP003040), *Fusobacterium mortiferum* OM06-15BH (accession no. NZ_QSTZ01000001), *Flavonifractor plautii* 2789STDY5834892 (accession no. CZAS01000006), *Anaerostipes caccae* NBRC 114412 (accession no. CP084016), *Eubacterium callanderi* AHG0017 (accession no. NZ_QYRZ01000037), *Megasphaera elsdenii* indica sctg_0022_0001 (accession no. NQMW01000022), *Coprococcus catus* AM28-39 (accession no. NZ_QVFD01000002), *Clostridium symbiosum* ATCC 14940 (accession no. AWSU01000039), *Eubacterium limosum* ATCC 8486 (accession no. CP019962), *Butyricicoccus pullicaecorum* An179 (accession no. NZ_NFKL01000012), and *Anaerotruncus colihominis* ERR3414572 (accession no. NZ_CAKNJW010000218) were obtained from the National Center for Biotechnology Information (https://www.ncbi.nlm.nih.gov/) database and aligned using GENETYX ver. 14 (Genetyx Co., Tokyo, Japan). Based on the alignment results, target sites for *but* detection were identified, and the primer set, but_652F3 (5′-CARCTBGGHATYGGBGGWATGCCHAAYGC-3′) and but_1025R3 (5′-GCDCCBADVACRAARTCNARCTGWCCRCC-3′), was constructed (Table S3). An annealing temperature of 63°C and a primer concentration of 1.0 µM were found to be optimal. The amplicon size was 395–401 bp.

### Preparation of standard DNA

DNA extracted from a pure culture of *Anaerobutyricum hallii* YIT 10064^T^ was diluted to a concentration equivalent to 2 × 10^7^ cells/mL based on the bacterial count determined using 4′,6-diamidino-2-phenylindole staining (52). Using the diluted DNA as a template, the target DNA was amplified by PCR with Takara Taq (Takara, #R001AM) and the newly developed primer set for *but*. The 40 µL reaction mixture was composed of 10× PCR buffer (no MgCl_2_), dNTP (2.5 mM each), a 25 mM MgCl_2_ solution, water for injection (Thermo Fisher Scientific Inc., #AM9937), each specific primer, Taq polymerase (5 units/µL), and 1 µL of template DNA. Agarose gel electrophoresis confirmed the presence of a single band of the desired size. The amplified product was purified using an HP PCR Product Purification Kit (Nihon Genetics, Tokyo, Japan, #11732668001), following the manufacturer’s instructions. OD_260_ was measured using a DU730 spectrophotometer (Beckman Coulter Inc., Brea, CA, USA), and the DNA concentration was calculated based on the measured OD_260_ value. The purified DNA product was ligated with a T-Vector pMD20 (Takara, Shiga, Japan, #3270) using the DNA Ligation Kit ver. 2.1 (Takara, #6022) and transformed into XL10-Gold Ultracompetent Cells (Agilent, Santa Clara, CA, USA, #200314). Colonies with target DNA incorporated into the plasmid vector were selected using colony PCR, and the plasmids were extracted using the QIAprep Spin Miniprep Kit (Qiagen, Hilden, Germany, #27106). The concentration of the extracted DNA was measured using the PicoGreen dsDNA Assay Kit (Thermo Fisher Scientific Inc., Waltham, MA, USA, #P7589). The DNA was adjusted to a concentration of 2 × 10^10^ copies/μL and used as the standard DNA in the subsequent qPCR experiments; the DNA was stored at −30°C until further use.

### Preparation of standard RNA

The T7 promoter sequence (TAATACGACTCACTATATAGGGAGA) was added to the 5′ end of but_652F3; PCR was performed using a primer pair with the T7 tagged forward primer and but_1025R3, and *Anaerobutyricum hallii* YIT 10064^T^ was used as template DNA. After purification and the measurement of the DNA concentration as described above, 0.2 µg of purified DNA product was used as a template to perform *in vitro* transcription and DNase treatment using the MEGAscript T7 Transcription Kit (Thermo Fisher Scientific Inc., #AM1333). After purifying the RNA obtained using the MEGAclear Transcription Clean-Up Kit (Thermo Fisher Scientific Inc., #AM1908), OD_260_ was measured to determine the concentration. RNA was adjusted to a concentration of 2 × 10^10^ copies/μL and used as the standard RNA in the subsequent RT-qPCR experiments; the RNA was stored at −80°C until use. DNA and RNA concentrations were determined using the following equation:


$$Concentration\;(pmol/mL)=OD_{260}\times 100/(1.5N_{A}+0.71N_{C}+1.20N_{G}+0.84N_{T(U)})$$

where N_A_: 126, N_C_: 56, N_G_: 104, and N_T(U)_: 115.

### DNA extraction and qPCR

A fresh culture of each bacterial strain (200 µL) was centrifuged at 13,000 × *g* for 10 min, and the supernatant was discarded. The pellet was recovered and stored at −30°C until DNA extraction. Fecal samples were weighed and suspended in nine volumes of phosphate-buffered saline, and 200 µL of the fecal suspension was stored at −30°C until use for DNA extraction. DNA was extracted as previously described (53). The 10 µL reaction mixture was composed of 10× PCR buffer (no MgCl_2_), dNTP (2.5 mM each), a 25 mM MgCl_2_ solution, water for injection (Thermo Fisher Scientific Inc.), a 1:300 dilution of SYBR green I (Lonza, Basel, Switzerland, #50513), 50× ROX Reference Dye (Thermo Fisher Scientific Inc., #12223012), each specific primer at a 1.0 µM concentration, Taq polymerase (5 units/µL), 1.1 µg/µL Taq start antibody (Takara, #Z9251N), and 5 µL of template DNA. The newly developed *but*-specific primers, but_652F3/but_1025R3, were used. Amplification and detection were performed in 384-well optical plates (Sarstedt AG & Co. KG, Nümbrecht, Germany, #72.1984.202) using the ABI PRISM 7900HT system (Thermo Fisher Scientific Inc.). The reaction mixture was incubated at 94°C for 5 min, followed by 40 cycles at 94°C for 20 s, 63°C for 20 s, and 72°C for 34 s. A melting curve was established to distinguish the target PCR products from non-target ones through slow heating from 60 to 95°C at a rate of 0.2°C/s with continuous fluorescence measurement. Using the primer sets previously published in the studies by Louis and Flint (24), Trachsel et al. (25), and Wang et al. (26) (Table S3), qPCR was performed under the reaction conditions described in each study. To establish a standard curve, 10^1^–10^5^ standard DNA copies were used per reaction. *C_q_* values in the linear range of the assay were applied to the analytical curve generated within the same experiment to obtain the corresponding copy numbers in each nucleic acid sample; these were converted to counts per milliliter of culture or per gram of feces.

### Total RNA extraction and RT-qPCR

For RNA stabilization, two volumes of the RNAprotect Bacteria Reagent (Qiagen, #76506) were added to the samples. After incubation for 10 min at 25°C, the samples were centrifuged at 13,000 × *g* for 10 min. The supernatant was discarded, and the pellet was recovered and stored at −80°C until RNA extraction. RNA was extracted as previously described (54). *but* expression levels were measured using two-step RT-qPCR. First, RNA was subjected to genomic DNA removal and reverse transcription using the PrimeScript RT Reagent Kit with the gDNA Eraser (Takara, #RR047A). Second, qPCR was performed as described above. For RT-qPCR, 10^2^–10^7^ standard RNA copies were used per reaction to establish a standard curve. *C_q_* values in the linear range of the assay were applied to the analytical curve generated within the same experiment to obtain the corresponding copy numbers in each nucleic acid sample; these values were converted to counts per milliliter of culture or per gram of feces. *but* expression levels were measured using the newly designed primer set, but_652F3/but_1025R3. The number of viable bacteria in the intestinal microbiota and *in vitro* culture milieux, except for those of LcS and BbrY, was determined using a one-step RT-qPCR system targeting 16S or 23S rRNA as previously described (54–56). LcS and BbrY cell numbers were determined using qPCR as previously described (57, 58). The primers used for these analyses are listed in Table S4.

### Determination of primer specificity

DNA fractions extracted from representative bacterial strains harboring *but* (20 strains) and those devoid of *but* (64 strains), at a dose corresponding to 10^5^ cells, were assessed through qPCR using the newly developed *but*-specific primers and previously reported primers. Using the standard curve for *but* DNA, the amplified signal was judged to be positive (+) when it was more than 10^4^ standard copies per reaction and negative (−) when it was less than 10^4^ standard copies per reaction.

### Determination of primer sensitivity

The detection sensitivity of the newly designed primer set was evaluated using the standard DNA and RNA as described above. Serial standard DNA and RNA dilutions corresponding to copy numbers ranging from 10^1^ to 10^5^ and 10^2^ to 10^7^ copies per reaction were assessed using qPCR and RT-qPCR assays, respectively. The lower copy number limit at which quantitative analysis was possible was determined by assessing the correlation between the *C_q_* value obtained and standard DNA and RNA copy numbers. The range of DNA and RNA concentrations at which there was linearity with the *C_q_* value was confirmed (Fig. S1).

### Measurement of SCFA concentration and pH

The concentrations of SCFAs (acetate, butyrate, and propionate) in feces and the *in vitro* culture milieux were measured using a Waters HPLC system (Waters 432 Conductive Detector, Waters, Milford, MA, USA) and Shodex Rspak KC-811 column (Showa Denko, Tokyo, Japan) as previously described (59). For the *in vitro* experiment, the change in butyrate concentration per hour (Δbutyrate production) was calculated by subtracting the butyrate concentration in the previously collected sample from that in the sample collected at the time, and the values were divided by the time interval. Fecal pH was measured through the direct insertion of a D-51 pH meter (Horiba Seisakusho, Kyoto, Japan) into the samples (59).

### *In vitro* mixed culture experiments

*Anaerostipes hadrus* YIT 13225, *Anaerobutyricum hallii* YIT 10064^T^, *Roseburia intestinalis* YIT 10172^T^ (strains harboring *but*), and *Prevotella copri* YIT 12933^T^ (a strain not harboring *but*) bacterial solutions were centrifuged at 7,740 × *g* for 10 min, and the supernatant was discarded. An equal volume of modified Gifu Anaerobic Medium (GAM) broth (Shimadzu, Kyoto, Japan, #05433) supplemented with 1% glucose (Kanto Chemical Co., Inc., Tokyo, Japan, #10017-00) was added to the pellet and mixed thoroughly. The washed bacterial solution was diluted 10-fold with modified GAM broth supplemented with 1% glucose and adjusted to an OD_600_ of 0.1. One hundred microliters of each bacterial solution was simultaneously inoculated into a tube containing 10 mL of modified GAM broth supplemented with 1% glucose. The bacterial mixture was cultured at 37°C and sampled at 0, 6, 8, 10, 12, 14, and 24 h. The samples were subjected to SCFA analysis, qPCR, and RT-qPCR. The procedures were performed in an anaerobic glove box.

### Analysis of changes in fecal *but* amount and *but* expression levels

Fecal samples were acquired from 10 healthy male adults (age: 38.5 ± 13.0 years [mean ± SD]) who received one packet of Synprotec (formerly called Super Synbiotics LBG-P, Yakult Honsha Co., Ltd., Tokyo, Japan) thrice daily. This product contains at least 1  × 10^8^ CFU/packet of live LcS and live BbrY as probiotics and 6.8 g/packet of galacto-oligosaccharides as prebiotics. The study period, which spanned non-intake of synbiotics (1 week), intake of synbiotics (2 weeks), and discontinuation of intake (2 weeks), was 5 weeks. Intake of other probiotics and prebiotics was prohibited during the study period. Fecal samples were collected on the day before the start of synbiotic intake (0 weeks), 1 and 2 weeks after intake (1 and 2 weeks), and 2 weeks after the discontinuation of intake (4 weeks) (Fig. S4).

### Statistical analyses

Statistical analyses were performed using R (ver. 4.0.5) (Posit PBC, Boston, MA, USA). Pearson’s correlation coefficient was used to analyze the relationships between items. The non-parametric Wilcoxon signed-rank test was used to compare fecal *but* abundance, *but* expression levels, viable bacterial counts, SCFA concentration, and pH before and after synbiotic intake. *P* values <0.05 were considered statistically significant. Data are presented as the mean and standard deviation of the values obtained in the representative experiments.

## Data Availability

All data are available in the article and supplemental material.

## SUPPLEMENTAL MATERIAL

Supplemental material aem.01836-25-s0001.docx Tables S1 to S4; Fig. S1 to S4.

ASM does not own the copyrights to Supplemental Material that may be linked to, or accessed through, an article. The authors have granted ASM a non-exclusive, world-wide license to publish the Supplemental Material files. Please contact the corresponding author directly for reuse.
