# Supplementary material for: A reverse transcription-quantitative polymerase chain reaction system for evaluating intestinal butyrate production by fecal bacteria
Source: Appl Environ Microbiol. 2025 Dec 12;92(1):e01836-25. doi: 10.1128/aem.01836-25 (PMC12838319; doi:10.1128/aem.01836-25)
Supplement: Supplemental material — Tables S1 to S4; Fig. S1 to S4. [file aem.01836-25-s0001.docx]

**Supplemental Materials**

**Supplementary Table 1. Effects of synbiotic intake on gut microbiota**

|  | 0 weeks | | 1 week | | 2 weeks | | 4 weeks | |
| --- | --- | --- | --- | --- | --- | --- | --- | --- |
| **Total bacteria** | 10.9 ± 0.2 | 100 | 11.1 ± 0.2 | 100 | 11.1 ± 0.3 | 100 | 11.0 ± 0.2 | 100 |
| **Total predominant obligate anaerobes** | 10.7 ± 0.3 | 100 | 10.8 ± 0.2 | 100 | 10.9 ± 0.3 | 100 | 10.7 ± 0.3 | 100 |
| *Clostridium coccoides* group | 10.1 ± 0.3 | 100 | 10.0 ± 0.2 | 100 | 10.1 ± 0.5 | 100 | 10.2 ± 0.2 | 100 |
| *C. leptum* subgroup | 10.0 ± 0.5 | 100 | 10.0 ± 0.3 | 100 | 10.0 ± 0.8 | 100 | 10.1 ± 0.4 | 100 |
| *Bacteroides fragilis* group | 9.7 ± 0.6 | 100 | 9.5 ± 0.7 | 100 | 9.5 ± 0.9 | 100 | 9.8 ± 0.6 | 100 |
| *Bifidobacterium* | 10.0 ± 0.6 | 100 | 10.5 ± 0.2* | 100 | 10.6 ± 0.3** | 100 | 9.8 ± 0.7^††^ | 100 |
| **Other obligate anaerobes** |  |  |  |  |  |  |  |  |
| *Atopobium* cluster | 9.4 ± 0.5 | 100 | 9.5 ± 0.4 | 100 | 9.5 ± 0.6 | 100 | 9.4 ± 0.4 | 100 |
| *Prevotella* | 9.7 ± 1.5 | 80 | 9.5 ± 0.4 | 90 | 9.5 ± 0.6 | 80 | 9.4 ± 2.0 | 90 |
| *C. difficile* | <2.3 | 0 | <2.3 | 0 | <2.3 | 0 | <2.3 | 0 |
| *C. perfringens* | 4.2 ± 1.2 | 70 | 4.4 ± 1.3 | 60 | 3.5 ± 1.0* | 60 | 4.0 ± 1.1 | 30 |
| **Facultative anaerobes** |  |  |  |  |  |  |  |  |
| Total lactobacilli ^a^ | 5.1 ± 1.4 | 100 | 7.0 ± 0.3** | 100 | 7.2 ± 0.5** | 100 | 4.7 ± 1.3^††^ | 90 |
| *Enterobacteriaceae* | 6.0 ± 1.2 | 80 | 6.2 ± 1.1 | 90 | 6.1 ± 1.2 | 90 | 6.8 ± 1.2 | 90 |
| *Enterococcus* | 5.5 ± 1.1 | 80 | 5.4 ± 1.4 | 80 | 5.9 ± 1.5 | 80 | 4.9 ± 1.2^†^ | 80 |
| *Streptococcus* | 8.4 ± 0.5 | 100 | 8.4 ± 0.4 | 100 | 8.5 ± 0.5 | 100 | 8.6 ± 0.5 | 100 |
| *Staphylococcus* | 4.6 ± 1.2 | 100 | 4.2 ± 0.3 | 90 | 4.4 ± 1.0 | 90 | 4.6 ± 1.2 | 90 |
| **Aerobes** |  |  |  |  |  |  |  |  |
| *Pseudomonas* | 5.6 | 10 | 5.2 | 20 | 3.9 | 20 | 3.9± 0.9 | 40 |
| LcS ^b^ | <4.9 | 0 | 7.2 ± 0.4 | 100** | 7.3 ± 0.5 | 100** | <4.9 | 0 ^††^ |
| BbrY ^b^ | <4.9 | 0 | 6.1 ± 0.5 | 100** | 6.2 ± 0.3 | 100** | <4.9 | 0 ^††^ |

Results are expressed as Log_10_ cells/g feces (mean ± standard deviation [SD]) and detection rate (%).

^a^ Total lactobacilli represents the sum of the following genera and species: *Lactobacillus* (previously *Lactobacillus gasseri* subgroup) and *Levilactobacillus brevis* (previously *Lactobacillus brevis*), *Lacticaseibacillus* (previously *Lactobacillus casei* subgroup), *Limosilactobacillus fermentum* (previously *Lactobacillus fermentum*), *Fructilactobacillus fructivorans* (previously *Lactobacillus fructivorans*), *Lactiplantibacillus* (previously *Lactobacillus plantarum* subgroup), *Limosilactobacillus* (previously *Lactobacillus reuteri* subgroup),　*Liquorilactobacillus* and *Ligilactobacillus* (previously *Lactobacillus ruminis* subgroup), and *Latilactobacillus* (previously *Lactobacillus sakei* subgroup).

^b^ Bacterial counts of the administered bacteria (LcS and BbrY) were quantified by qPCR.

^*^ *P* < 0.05, ^**^ *P* < 0.01: Comparison before and after intake (Wilcoxon signed-rank test, Fisher’s direct probability test)

^†^ *P* < 0.05, ^††^ *P* < 0.01: Comparison between 2 and 4 weeks (Wilcoxon signed-rank test, Fisher’s direct probability test)

**Supplementary Table 2. Bacterial strains and culture conditions**

| Taxon | Strain | Medium | Gas phase | Temperature |
| --- | --- | --- | --- | --- |
| *Roseburia faecis* | YIT 11921^T^ | Modified GAM broth + 1% glucose | anaerobic | 37 ℃ |
| *Roseburia intestinalis* | YIT 10172^T^ | Modified GAM broth + 1% glucose | anaerobic | 37 ℃ |
| *Roseburia hominis* | JCM 17582 | YCFA broth | anaerobic | 37 ℃ |
| *Butyricicoccus pullicaecorum* | YIT 12785^T^ | Modified PY broth | anaerobic | 37 ℃ |
| *Anaerostipes hadrus* | YIT 13225 | Modified GAM broth + 1% glucose | anaerobic | 37 ℃ |
| *Agathobacter rectalis* | YIT 6082^T^ | Modified GAM broth + 1% glucose | anaerobic | 37 ℃ |
| *Faecalibacterium prausnitzii* | YIT 10067^T^ | Medium for *F. prausnitzii* | anaerobic | 37 ℃ |
| *Anaerobutyricum hallii* | YIT 10064^T^ | Modified GAM broth + 1% glucose | anaerobic | 37 ℃ |
| *Roseburia inulinivorans* | YIT 11922^T^ | Modified GAM broth + 1% glucose | anaerobic | 37 ℃ |
| *Coprococcus eutactus* | YIT 10160^T^ | Modified GAM broth + 1% glucose | anaerobic | 37 ℃ |
| *Anaerostipes caccae* | YIT 10168^T^ | Modified GAM broth + 1% glucose | anaerobic | 37 ℃ |
| *Eubacterium callanderi* | YIT 10175^T^ | Modified GAM broth + 1% glucose | anaerobic | 37 ℃ |
| *Megasphaera elsdenii* | YIT 6063^T^ | Modified GAM broth + 1% glucose | anaerobic | 37 ℃ |
| *Clostridium symbiosum* | YIT 11480^T^ | Modified GAM broth + 1% glucose | anaerobic | 37 ℃ |
| *Eubacterium limosum* | YIT 6067^T^ | Modified GAM broth + 1% glucose | anaerobic | 37 ℃ |
| *Anaerostipes butyraticus* | YIT 12362^T^ | Medium for *F. prausnitzii* | anaerobic | 37 ℃ |
| *Coprococcus catus* | YIT 11484^T^ | Modified GAM broth + 1% glucose | anaerobic | 37 ℃ |
| *Fusobacterium mortiferum* | YIT 10361^T^ | Modified GAM broth + 1% glucose | anaerobic | 37 ℃ |
| *Flavonifractor plautii* | YIT 12796^T^ | Modified GAM broth + 1% glucose | anaerobic | 37 ℃ |
| *Anaerotruncus colihominis* | JCM 15631 | Modified GAM broth + 1% glucose | anaerobic | 37 ℃ |
| *Blautia luti* | YIT 12257^T^ | Modified GAM broth + 1% glucose | anaerobic | 37 ℃ |
| *Bifidobacterium pseudocatenulatum* | YIT 4072^T^ | Modified GAM broth + 1% glucose | anaerobic | 37 ℃ |
| *Bifidobacterium adolescentis* | YIT 4011^T^ | Modified GAM broth + 1% glucose | anaerobic | 37 ℃ |
| *Bacteroides vulgatus* | YIT 6159^T^ | Modified GAM broth + 1% glucose | anaerobic | 37 ℃ |
| *Prevotella copri* | YIT 12933^T^ | Modified GAM broth + 1% glucose | anaerobic | 37 ℃ |
| *Ruminococcus bromii* | YIT 6078 | Modified GAM broth + 1% glucose | anaerobic | 37 ℃ |
| *Akkermansia muciniphila* | YIT 11774^T^ | Modified GAM broth + 1% glucose | anaerobic | 37℃ |
| *Parabacteroides distasonis* | YIT 12678 | Modified GAM broth + 1% glucose | anaerobic | 37 ℃ |
| *Bifidobacterium breve* | YIT 4014^T^ | Modified GAM broth + 1% glucose | anaerobic | 37 ℃ |
| *Bifidobacterium bifidum* | YIT 4039^T^ | Modified GAM broth + 1% glucose | anaerobic | 37 ℃ |
| *Eggerthella lenta* | YIT 6077^T^ | Modified GAM broth + 1% glucose | anaerobic | 37℃ |
| *Eubacterium siraeum* | YIT 10049^T^ | Modified GAM broth + 1% glucose | anaerobic | 37 ℃ |
| *Bifidobacterium dentium* | YIT 4017^T^ | Modified GAM broth + 1% glucose | anaerobic | 37 ℃ |
| *Bifidobacterium animalis* | YIT 4044^T^ | Modified GAM broth + 1% glucose | anaerobic | 37 ℃ |
| *Veillonella atypica* | YIT 6081^T^ | GAM broth + 1.5%Na-Lactate | anaerobic | 37 ℃ |
| *Streptococcus mutans* | YIT 2026^T^ | Modified GAM broth + 1% glucose | aerobic | 37 ℃ |
| *Bifidobacterium angulatum* | YIT 4012^T^ | Modified GAM broth + 1% glucose | anaerobic | 37 ℃ |
| *Lactobacillus gasseri* | YIT 0192^T^ | Modified GAM broth + 1% glucose | anaerobic | 37 ℃ |
| *Streptococcus anginosus* | YIT 11237^T^ | Modified GAM broth + 1% glucose | anaerobic | 37 ℃ |
| *Raoultella planticola* | YIT 10131^T^ | Modified GAM broth + 1% glucose | aerobic | 37 ℃ |
| *Veillonella parvula* | YIT 6072^T^ | GAM broth + 1.5%Na-Lactate | anaerobic | 37 ℃ |
| *Clostridium asparagiforme* | YIT 12840^T^ | Modified GAM broth + 1% glucose | anaerobic | 37 ℃ |
| *Clostridium hylemonae* | YIT 12258^T^ | Modified GAM broth + 1% glucose | anaerobic | 37 ℃ |
| *Bifidobacterium pseudolongum* | YIT 4102^T^ | Modified GAM broth + 1% glucose | anaerobic | 37 ℃ |
| *Bifidobacterium gallinarum* | YIT 4094^T^ | Modified GAM broth + 1% glucose | anaerobic | 37 ℃ |
| *Citrobacter amalonaticus* | YIT 10116^T^ | Modified GAM broth + 1% glucose | aerobic | 37 ℃ |
| *Atopobium minutum* | YIT 0194 | Modified GAM broth + 1% glucose | anaerobic | 37 ℃ |
| *Enterobacter cloacae* | YIT 6041^T^ | Modified GAM broth + 1% glucose | aerobic | 37 ℃ |
| *Ruminococcus torques* | YIT 10159^T^ | Modified GAM broth + 1% glucose | anaerobic | 37 ℃ |
| *Bifidobacterium catenulatum* | YIT 4072^T^ | Modified GAM broth + 1% glucose | anaerobic | 37 ℃ |
| *Dialister succinatiphilus* | YIT 11850^T^ | Modified GAM agar^H^ + 1% glucose | anaerobic | 37 ℃ |
| *Bifidobacterium longum* | YIT 4021^T^ | Modified GAM broth + 1% glucose | anaerobic | 37 ℃ |
| *Collinsella aerofaciens* | YIT 10235^T^ | Modified GAM broth + 1% glucose | anaerobic | 37 ℃ |
| *Bacteroides uniformis* | YIT 6164^T^ | Modified GAM broth + 1% glucose | anaerobic | 37 ℃ |
| *Ruminococcus gnavus* | YIT 6176^T^ | Modified GAM broth + 1% glucose | anaerobic | 37℃ |
| *Bacteroides stercoris* | YIT 12663 | Modified GAM broth + 1% glucose | anaerobic | 37 ℃ |
| *Bacteroides fragilis* | YIT 6158^T^ | Modified GAM broth + 1% glucose | anaerobic | 37 ℃ |
| *Bacteroides ovatus* | YIT 6161^T^ | Modified GAM broth + 1% glucose | anaerobic | 37 ℃ |
| *Phocaeicola plebeius* | YIT 12661 | Modified GAM broth + 1% glucose | anaerobic | 37 ℃ |
| *Bacteroides thetaiotaomicron* | YIT 6163^T^ | Modified GAM broth + 1% glucose | anaerobic | 37 ℃ |
| *Eubacterium ramulus* | YIT 12128^T^ | Modified GAM broth + 1% glucose | anaerobic | 37 ℃ |
| *Alistipes onderdonkii* | YIT 12691 | Modified GAM broth + 1% glucose | anaerobic | 37 ℃ |
| *Enterococcus avium* | YIT 10255^T^ | Modified GAM broth + 1% glucose | aerobic | 37 ℃ |
| *Enterocloster clostridioformis* | YIT 6051^T^ | Modified GAM broth + 1% glucose | anaerobic | 37 ℃ |
| *Alistipes finegoldii* | YIT 12685 | Modified GAM broth + 1% glucose | anaerobic | 37 ℃ |
| *Alistipes indistinctus* | YIT 12060^T^ | Modified GAM broth + 1% glucose | anaerobic | 37 ℃ |
| *Clostridium scindens* | YIT 6171^T^ | Modified GAM broth + 1% glucose | anaerobic | 37 ℃ |
| *Blautia producta* | YIT 6141^T^ | Modified GAM broth + 1% glucose | anaerobic | 37 ℃ |
| *Escherichia coli* | YIT6044^T^ | Modified GAM broth + 1% glucose | aerobic | 37 ℃ |
| *Clostridium citroniae* | YIT 12646^T^ | Modified GAM broth + 1% glucose | anaerobic | 37 ℃ |
| *Enterococcus faecalis* | YIT 2031^T^ | Modified GAM broth + 1% glucose | aerobic | 37 ℃ |
| *Citrobacter freundii* | YIT 6045^T^ | Modified GAM broth + 1% glucose | aerobic | 37 ℃ |
| *Olsenella uli* | YIT 12014^T^ | Modified GAM broth + 1% glucose | anaerobic | 37 ℃ |
| *Eubacterium ventriosum* | YIT 10066^T^ | Modified GAM+GCA broth | anaerobic | 37 ℃ |
| *Coprococcus comes* | YIT 12793^T^ | Modified GAM broth + 1% glucose | anaerobic | 37 ℃ |
| *Erysipelatoclostridium ramosum* | YIT 10062^T^ | Modified GAM broth + 1% glucose | anaerobic | 37 ℃ |
| *Odoribacter splanchnicus* | YIT 12675^T^ | Modified GAM broth + 1% glucose | anaerobic | 37 ℃ |
| *Fusobacterium varium* | YIT 12723 | Modified GAM broth + 1% glucose | anaerobic | 37 ℃ |
| *Citrobacter koseri* | YIT 10117^T^ | Modified GAM broth + 1% glucose | aerobic | 37 ℃ |
| *Acidaminococcus fermentans* | YIT 6071^T^ | Modified GAM broth + 1% glucose | anaerobic | 37 ℃ |
| *Subdoligranulum variabile* | YIT 12797^T^ | Modified GAM agar + 1% glucose | anaerobic | 37 ℃ |
| *Fusobacterium necrophorum subsp. necrophorum* | YIT 10343^T^ | Modified GAM broth + 1% glucose | anaerobic | 37 ℃ |
| *Fusobacterium nucleatum subsp.nucleatum* | YIT 6069^T^ | Modified GAM broth + 1% glucose | anaerobic | 37 ℃ |
| *Fusobacterium periodonticum* | YIT 12430^T^ | Modified GAM broth + 1% glucose | anaerobic | 37 ℃ |
| *Adlercreutzia equolifaciens* | YIT 12017^T^ | Modified GAM agar + 1% glucose | anaerobic | 37 ℃ |

GAM: Gifu Anaerobic Medium; YCFA: Yeast Casitone Fatty Acids; PY: Peptone Yeast Extract; GCA: Glucose Cellobiose Aminovalerate; ℃: degrees Celsius

**Supplementary Table 3. Primer sequences used for *but* detection**

| No. | Primer | Sequence (5′-3′) | Amplicon size  (bp) | Annealing Temp. (℃) | Reference |
| --- | --- | --- | --- | --- | --- |
| 1 | but_652F3 | CARCTBGGHATYGGBGGWATGCCHAAYGC | 401 | 63 | This study |
|  | but_1025R3 | GCDCCBADVACRAARTCNARCTGWCCRCC |  |  |  |
| 2 | BCoATscrF | GCIGAICATTTCACITGGAAYWSITGGCAYATG | 557 | 53 | [24] |
|  | BCoATscrR | CCTGCCTTTGCAATRTCIACRAANGC |  |  |  |
| 3 | funbut-FWD | CARYTIGGIATYGGIGGIATSCC | 380 | 53 | [25] |
|  | funbut-REV | TGTCCGCCIGYICCRSWRAT |  |  |  |
| 4 | BUT-F | GAACGGAGCMTGYYTVCAGC | 124 | 45 | [26] |
|  | BUT-R | ATCCACAAACGCATCBACRTAC |  |  |  |

**Supplementary Table 4. Primer sequences used for intestinal microbiota analysis**

| **Target** | **Primer** | **Sequence (5’–3’)** | **Reference** |
| --- | --- | --- | --- |
| *Clostridium coccoides* group | g-Ccoc-F | AAATGACGGTACCTGACTAA | [55] |
|  | g-Ccoc-R | CTTTGAGTTTCATTCTTGCGAA |  |
| *C. leptum* subgroup | sg-Clept-F | GCACAAGCAGTGGAGT | [55] |
|  | sg-Clept-R3 | CTTCCTCCGTTTTGTCAA |  |
| *Bacteroides fragilis* group | g-Bfra-F2 | AYAGCCTTTCGAAAGRAAGAT | [55] |
|  | g-Bfra-R | CCAGTATCAACTGCAATTTTA |  |
| *Bifidobacterium* | g-Bifid-F | CTCCTGGAAACGGGTGG | [55] |
|  | g-Bifid-R | GGTGTTCTTCCCGATATCTACA |  |
| *Atopobium* cluster | c-Atopo-F | GGGTTGAGAGACCGACC | [55] |
|  | c-Atopo-R | CGGRGCTTCTTCTGCAGG |  |
| *Prevotella* | g-Prevo-F | CACRGTAAACGATGGATGCC | [55] |
|  | g-Prevo-R | GGTCGGGTTGCAGACC |  |
| *C. difficile* | Cd-lsu-F | GGGAGCTTCCCATACGGGTTG | [56] |
|  | Cd-lsu-R | TTGACTGCCTCAATGCTTGGGC |  |
| *C. perfringens* | s-Clper-F | GGGGGTTTCAACACCTCC | [55] |
|  | ClPER-R | GCAAGGGATGTCAAGTGT |  |
| *Lactobacillus*  (previously *Lactobacillus gasseri* subgroup） | sg-Lgas-F | GATGCATAGCCGAGTTGAGAGACTGAT | [55] |
|  | sg-Lgas-R | TAAAGGCCAGTTACTACCTCTATCC |  |
| *Levilactobacillus brevis*  (previously *Lactobacillus brevis*) | s-Lbre-F | ATTTTGTTTGAAAGGTGGCTTCGG | [55] |
|  | s-Lbre-R | ACCCTTGAACAGTTACTCTCAAAGG |  |
| *Lacticaseibacillus*  (previously *Lactobacillus casei* subgroup) | sg-Lcas-F | ACCGCATGGTTCTTGGC | [55] |
|  | sg-Lcas-R | CCGACAACAGTTACTCTGCC |  |
| *Limosilactobacillus fermentum*  (previously *Lactobacillus fermentum*) | LFer-1 | CCTGATTGATTTTGGTCGCCAAC | [55] |
|  | LFer-2 | ACGTATGAACAGTTACTCTCATACGT |  |
| *Fructilactobacillus fructivorans* (previously *Lactobacillus fructivorans*) | s-Lfru-F | TGCGCCTAATGATAGTTGA | [55] |
|  | s-Lfru-R | GATACCGTCGCGACGTGAG |  |
| *Lactiplantibacillus*  (previously *Lactobacillus plantarum* subgroup) | sg-Lpla-F | CTCTGGTATTGATTGGTGCTTGCAT | [55] |
|  | sg-Lpla-R | GTTCGCCACTCACTCAAATGTAAA |  |
| *Limosilactobacillus*  (previously *Lactobacillus reuteri* subgroup) | sg-Lreu-F | GAACGCAYTGGCCCAA | [55] |
|  | sg-Lreu-R | TCCATTGTGGCCGATCAGT |  |
| *Liquorilactobacillus and Ligilactobacillus*  (previously *Lactobacillus ruminis* subgroup) | sg-Lrum-F | CACCGAATGCTTGCAYTCACC | [55] |
|  | sg-Lrum-R | GCCGCGGGTCCATCCAAAA |  |
| *Latilactobacillus*  (previously *Lactobacillus sakei* subgroup) | sg-Lsak-F | CATAAAACCTAMCACCGCATGG | [55] |
|  | sg-Lsak-R | TCAGTTACTATCAGATACRTTCTTCTC |  |
| *Enterobacteriaceae* | En-lsu3F | TGCCGTAACTTCGGGAGAAGGCA | [55] |
|  | En-lsu3R | TCAAGGCTCAATGTTCAGTGTC |  |
| *Enterococcus* | g-Encoc-F | ATCAGAGGGGGATAACACTT | [55] |
|  | g-Encoc-R | ACTCTCATCCTTGTTCTTCTC |  |
| *Streptococcus* | g-Str-F | AGCTTAGAAGCAGCTATTCATTC | [57] |
|  | g-Str-R | GGATACACCTTTCGGTCTCTC |  |
| *Staphylococcus* | g-Staph-F | TTTGGGCTACACACGTGCTACAATGGACAA | [55] |
|  | g-Staph-R | AACAACTTTATGGGATTTGCWTGA |  |
| *Pseudomonas* | PSD7F | CAAAACTACTGAGCTAGAGTACG | [55] |
|  | PSD7R | TAAGATCTCAAGGATCCCAACGGCT |  |
| LcS | pLcS-57F | CTCAAAGCCGTGACGGTC | [58] |
|  | pLcS-597R | CACTAGGATTATTAGCACCACGT |  |
| BbrY | pBbrY-F | ATGGCAAAACCGGGCTGAA | [59] |
|  | pBbrY-R | GCGGATGAGAGGTGGG |  |

**Supplementary Figure 1. Detection limits for *but* mRNA and DNA as determined through reverse transcription–quantitative polymerase chain reaction (RT-qPCR) or qPCR**

Serial standard RNA and DNA dilutions corresponding to copy numbers ranging from 10^2^ to 10^7^ and 10^1^ to 10^5^ copies per reaction were assessed through RT-qPCR (left) and qPCR (right), respectively. The *Cq* values obtained were plotted against the log_10_ number of copies subjected to each reaction. Data are presented as the mean ± SD (A: n=5, B: n=12).

**Supplementary Figure 2. Temporal change in viable cell count in the mixed culture**

The combined viable counts of the *Clostridium coccoides* group and *Prevotella* as determined through RT-qPCR are shown (mean ± SD).

**Supplementary Figure 3. Correlation between fecal *but* copy number and concentrations of butyrate, propionate, and acetate**

Correlation plots between *but* copy number and butyrate (A), propionate (B), and acetate (C) concentrations for 40 fecal samples from healthy adults.

**Supplementary Figure 4. Schematic representation of the schedule for the pilot synbiotic intake study**
